# *E6* and *E7* Gene Polymorphisms in Human Papillomavirus Types-58 and 33 Identified in Southwest China

**DOI:** 10.1371/journal.pone.0171140

**Published:** 2017-01-31

**Authors:** Zuyi Chen, Yaling Jing, Qiang Wen, Xianping Ding, Tao Wang, Xuemei Mu, Yuwei Chenzhang, Man Cao

**Affiliations:** 1 Key Laboratory of Bio-Resources and Eco-Environment, Ministry of Education, Sichuan University, Chengdu, China; 2 Bio-resource Research and Utilization Joint Key Laboratory of Sichuan and Chongqing, Sichuan and Chongqing, China; 3 Institute of Medical Genetics, College of Life Science, Sichuan University, Chengdu, China; Fondazione IRCCS Istituto Nazionale dei Tumori, ITALY

## Abstract

Cancer of the cervix is associated with infection by certain types of human papillomavirus (HPV). The gene variants differ in immune responses and oncogenic potential. The *E6* and *E7* proteins encoded by high-risk HPV play a key role in cellular transformation. HPV-33 and HPV-58 types are highly prevalent among Chinese women. To study the gene intratypic variations, polymorphisms and positive selections of HPV-33 and HPV-58 *E6/E7* in southwest China, HPV-33 (*E6*, *E7*: n = 216) and HPV-58 (*E6*, *E7*: n = 405) *E6* and *E7* genes were sequenced and compared to others submitted to GenBank. Phylogenetic trees were constructed by Maximum-likelihood and the Kimura 2-parameters methods by MEGA 6 (Molecular Evolutionary Genetics Analysis version 6.0). The diversity of secondary structure was analyzed by PSIPred software. The selection pressures acting on the *E6*/*E7* genes were estimated by PAML 4.8 (Phylogenetic Analyses by Maximun Likelihood version4.8) software. The positive sites of HPV-33 and HPV-58 *E6/E7* were contrasted by ClustalX 2.1. Among 216 HPV-33 *E6* sequences, 8 single nucleotide mutations were observed with 6/8 non-synonymous and 2/8 synonymous mutations. The 216 HPV-33 *E7* sequences showed 3 single nucleotide mutations that were non-synonymous. The 405 HPV-58 *E6* sequences revealed 8 single nucleotide mutations with 4/8 non-synonymous and 4/8 synonymous mutations. Among 405 HPV-58 *E7* sequences, 13 single nucleotide mutations were observed with 10/13 non-synonymous mutations and 3/13 synonymous mutations. The selective pressure analysis showed that all HPV-33 and 4/6 HPV-58 *E6/E7* major non-synonymous mutations were sites of positive selection. All variations were observed in sites belonging to major histocompatibility complex and/or B-cell predicted epitopes. K93N and R145 (I/N) were observed in both HPV-33 and HPV-58 *E6*.

## Introduction

Cervical cancer is the third most common cancer in women worldwide, and a persistent infection of the high-risk human papillomavirus (HPV) is a major risk factor for cervical cancer [1,2]. Approximately 500000 new cases of cervical cancer are diagnosed every year, and the disorder causes 250000 deaths; more than 85% of all patients are from low-income countries [3,4]. The risk of developing cervical cancer in HPV-infected patients is 50-fold higher than uninfected women [5,6]. Genital HPV types are typically divided into two groups according to their presumed oncogenic potential. HPV-16, 18, 31, 33, 35, 39, 45, 51, 52, 53, 56, 58, and 59 are the common high oncogenic risk types [7].

The early expressing proteins *E6* and *E7* of high-risk HPV are the primary oncoproteins involved in human epithelial cell immortalization and transformation, and act through their interactions with numerous host proteins [8]. A primary target of *E6* is the p53 tumor suppressor protein. Additionally, *E6* activates telomerase expression and modulates the activities of PDZ domain-containing proteins and tumor necrosis factor receptors [9,10]. The *E7* proteins have evolved as a primary target of the retinoblastoma (Rb) family of proteins that control the activity of E2F transcription factors for degradation, resulting in the increase in the expression of E2F-responsive genes [11]. Moreover, knockdown of *E6* and *E7* expression in cervical cancer cells successfully suppresses cell growth and induces apoptosis [12]. Thus, HPV *E6*/*E7* can form an ideal target for diagnostic detection and therapeutic vaccine design.

Worldwide, HPV-33 and HPV-58 account for approximately 5% and 2% of cervical cancer cases, respectively; nevertheless, these HPV types show remarkably high infection rates in East Asia [13–16]. The areas of China, where cases of carcinoma *in situ* and cervical cancer caused by HPV-58 were more than the cases caused by HPV-18, were rated second; while HPV-33 was ranked third or fourth [17–19]. Furthermore, in our previous study, we reported that the detection rates of HPV-16 and 18 had decreased and that of HPV-58 and HPV-33 had increased over a 6-year period [20]. Therefore, researchers in China should devote greater attention to the more prevalent high-risk types of HPV-58 and HPV-33 for vaccine design than areas HPV-58 and HPV-33 are not so common.

The distribution and variation of HPV types show some degree of ethnic and geographical differences; consequently, the diagnosis, treatment, and ideal vaccine constructs necessarily need to be local [21,22]. Amino acid changes of high-risk HPV *E6*/*E7* might affect carcinogenic potential, host immune responses, and therapeutic effects of the vaccine. Among different types of HPV, there are types and variants that can acquire biological advantages through fixed mutations in their genomes, and even small variations could result in small adaptive improvements, possibly altering the composition of a HPV population [23]. This study may contribute more information in understanding HPV distribution by contrasting the HPV-33 and HPV-58 positive selections. Unfortunately, the data available on HPV-33 and HPV-58 *E6*/*E7* are still limited, especially in China. Therefore, the present study aimed to investigate the HPV-33 and HPV-58 *E6*/*E7* gene polymorphisms, intratypic variations and positive selections in southwest China by examining a large series of samples covering the Chinese population. This study can provide essential data for future research on viral prevention and therapeutics. Above all, our findings may offer critical information for developing diagnostic probes and vaccines, specifically designed based on HPV-33 and HPV-58 *E6*/*E7*.

## Materials and Methods

### Ethics statement

The study was approved by the Education and Research Committee and the Ethics Committee of Sichuan University, Sichuan, China. Before the samples were collected, a written informed consent was obtained from all the patients or their guardians, and patient/study subject privacy was carefully protected.

### Study population and specimen collection

Between January 1, 2009 and December 31, 2015, 16793 (age range 16–87, average age 33.02, median age 29) cervical specimens were collected from women who underwent cervical screenings, histology and cytology evaluations for cervical diseases at Sichuan Reproductive Health Research Center Affiliated Hospital, The Angel Women’s and Children’s Hospital, The Chengdu Western Hospital Maternity Unit, The People’s Hospital of Pengzhou, and Chongqing The Fourth Hospital. Women over 14 years of age and with visible cervical lesions and/or HPV-related diseases (e.g., cervicitis, cervical intraepithelial neoplasia, and cervical cancer) were eligible for inclusion [20]. Specimens were collected from the participants and stored in a preservative buffer (NaCl 9g, C_6_H_5_CO_2_Na 10g, H₂O 1L) at -20°C.

### Genomic DNA extraction and HPV typing

HPV-DNA was extracted and examined using the Human Papillomavirus Genotyping Kit For 23 Types (Yaneng Bio, Shenzhen, China) according to the manufacturer’s instructions. This kit enabled the classification of the 23 HPV types (HPV 16, 18, 31, 33, 35, 39, 45, 51, 52, 53, 56, 58, 59, 66, 68, 73, 83, MM4, 6, 11, 42, 43, and 44). 326 HPV-33 and 592 HPV-58 positive samples were collected.

### PCR amplification and identification of variants

The complete *E6*/*E7* genes of HPV-33 were first amplified by polymerase chain reaction (PCR) in the thermal cycler (Longgene, Hangzhou, China) using the primers: 5'-AAAAAAGTAGGGTGTAACCGA-3' and 5'-TGCCACTGTCATCTGCTGT-3'; this step was followed by a second round of amplification using the inner primers: 5'-ACGGTGCATATATAAAGCAAACATT-3' and 5'-CTTCTACCTCAAACCAACC-AGTACA-3', when needed. The HPV-58 *E6*/*E7* fragment was amplified using specific primers described previously [24]. Each 50 μL PCR reaction mixture contained 5 μL of extracted DNA (10–100 ng), 200 μmoL MgCl2 and dNTPs, 2 U of Pfu DNA polymerase (Sangon Biotech, Shanghai, China), and 0.25 μmoL of each primer. The cycling conditions employed were as follows: 95°C for 10 min; 35 cycles of 94°C for 50 sec, 54°C (different for each gene) for 60 sec, 72°C for 60 sec; and a final step of 72°C for 7 min. The PCR amplification products were visualized on 2% agarose gels stained with GeneGreen nucleic acid dye under the ultraviolet light WFH-202. Target products were sequenced at Sangon Biotech (Shanghai, China), and all the data were confirmed by repeating the PCR amplification and sequence analysis at least twice.

The sequences were subsequently analyzed by NCBI Blast and DNAMAN version5.2.2 (Lynnon Biosoft, Quebec, Canada). HPV-33 and HPV-58 DNA nucleotide positions were numbered according to their reference sequences M12732.1 and D90400, respectively. The E6/E7 sequences of 33HE01-33HE15 in HPV-33 and 58HE01-58HE17 in HPV-58 were published with the GenBank accession codes KX354744-KX354775.

### Sequence analysis

The secondary structures of the reference proteins were predicted by PSIPred server (http://bioinf.cs.ucl.ac.uk/psipred/) with the default parameters, which provided a simple and accurate secondary structure prediction method. Using a very stringent cross validation technique to evaluate the method’s performance, PSIPRED 3.2 achieves an average Q3 score of 81.6% [25]. The data were analyzed using SPSS version 19 (IBM, Armonk, NY, USA). The Pearson χ2 test was used to confirm the results. P < 0.05 was considered statistically significant. A mutation of which the frequency ≥ 10% was considered as a major mutation.

### Phylogenetic analysis of HPV-33 and HPV-58

Phylogenetic trees of respective HPV-33 *E6*/*E7* and HPV-58 *E6*/*E7* variation patterns were constructed through Maximum-likelihood trees by MEGA 6 using Kimura’s two-parameter model. The tree topology was evaluated by employing bootstrap resampled 1000 times [26]. The reference sequences used to construct the phylogenetic branches were collected from the GenBank sequence database and were listed in S1 and S2 Tables. HPV-33 and HPV-58 lineage and sublineage classification based on other unique *E6/E7* have been described in previous studies [24,27–36]. Numbers above the branches indicated the bootstrap values that are greater than 75%.

### Epitope prediction

ProPred-I server (http://www.imtech.res.in/raghava/propred1/) [37] was used to predict the human leukocyte antigen (HLA) class I binding promiscuous epitope(s) at the default settings (allele selection: all, threshold: 4%, tabular results: 4, proteasome filter: off, immunoproteasome: off). To predict epitope(s) for HLA class II alleles, ProPred software (http://www.imtech.res.in/raghava/propred/) [38] was used at the default settings (threshold %: 3.0, allele: all, display top scorers: blank). ABCpred server (http://www.imtech.res.in/raghava/abcpred/ABC_submission.html), which uses an artificial neural network, was used for the prediction of B-cell epitope(s) in the *E6* and *E7* sequences at the default settings (threshold value: 0.51, window length: 16; overlapping filter: no) [39].

### Selective pressure analysis and homology comparison

To estimate for positive selection at particular sites of the HPV-33 and HPV-58 *E6*/*E7* gene sequences, the *codeml* program in the PAML 4.8 was used. This program performed the likelihood ratio tests (LRTs) to infer nonsynonymous and synonymous nucleotide divergence for coding regions employing the method described by Nei and Gojobor [40–42]. HPV-33 and HPV-58 *E6/E7* protein sequences were aligned by ClustalX 2.1 (ftp://ftp.ebi.ac.uk/pub/software/clustalw2/) [43].

## Results

Among all the HPV-58 and HPV-33 samples, 405 sequences of HPV-58 *E6*/*E7* gene and 216 sequences of HPV-33 *E6*/*E7* gene were obtained owing to the small number of copies of infected HPV in some women and limited amplicons obtained for sequencing.

### Gene polymorphism of HPV-33 *E6*/*E7*

Compared with the HPV-33 reference sequence (GenBank: M12732.1), 104 of the 216 HPV-33 isolates (48.15%) showed complete *E6*/*E7* sequence homology with the reference and the remaining 112 (51.85%) isolates showed nucleotide variation.

Altogether, 11 nucleotide positions of *E6*/*E7* fragment showing sequence polymorphisms were identified among the 216 isolates. 8 biallelic mutations occurred in the 450-bp *E6* Open Reading Frame (ORF); more specifically, 6/8 were non-synonymous substitutions and 2/8 were synonymous mutations. In addition, two biallelic mutations and one triallelic (C706A/T) mutation were found over the 294-bp *E7* ORF, resulting in 4 amino acid changes of S29T, A45V, A45E, and Q97L (Table 1). The sequence variability of *E7* was lower than that of *E6*. The average probability of a nucleotide sequence deviation from the prototype was 20.37 substitutions per 10000 bp for *E6* and 15.28 substitutions per 10000 bp for *E7*. Mutations generating a frame shift or a premature stop codon were not observed.

**Table 1:** Nucleotide sequence mutation at *E6/E7* of 15 HPV 33 isolates.

sequence pattern	HPV33 *E6*								HPV33 *E7*			n (216)	sub-lineages
	2	2	3	3	3	4	5	5	6	7	8		
	1	7	2	6	8	4	4	4	5	0	6		
	3	3	9	4	7	6	2	9	8	6	2		
M12732	A	A	G	A	A	A	G	T	G	C	A		
33HE01	-	-	-	-	-	-	-	-	-	-	-	104	A1
33HE02	-	-	-	-	-	-	-	-	C	-	-	3	A1
33HE03	-	-	-	-	-	-	-	-	C	T	-	1	A1
33HE04	-	-	-	-	-	-	-	-	-	T	-	31	A1
33HE05	-	-	-	-	-	-	T	-	-	T	-	4	A1
33HE06	-	-	-	-	-	-	T	-	-	-	-	16	A1
33HE07	-	-	-	-	-	-	T	-	-	-	T	1	A1
33HE08	-	-	-	-	-	-	T	-	C	-	T	1	A1
33HE09	-	-	-	-	-	-	T	-	C	-	-	4	A1
33HE10	-	-	C	-	-	-	T	-	C	-	-	7	A1
33HE11	-	-	-	-	-	-	-	-	-	A	-	1	A1
33HE12	-	-	-	-	C	-	-	-	-	A	-	1	A1
33HE13	C	-	-	-	C	-	-	C	-	A	-	25	A1
33HE14	C	G	-	-	-	-	-	-	-	-	T	1	A3
33HE15	C	G	-	C	C	G	-	-	-	-	T	16	A3
aa mutation						Q	R						
	K		S	N	K	1	1		S	A	Q		
	3	-	7	8	9	1	4	-	2	4	9		
	5		4	6	3	3	5		9	5	7		
	N		T	H	N	R	I		T	V/E	L		
second structure	-	S	H	-	-	H	-	-	-	-	-		

The nucleotides conserved with respect to the reference sequence were marked with a dash (-), whereas a variation position was indicated by a letter. The “S” in the last row of the table means Sheet, the “H” means Helix. “n” represents the number of each sequence pattern among the 216 samples examined.

Two non-synonymous mutations (S74T and Q113R) occurred in the HPV-33 *E6* sequences encoding the alpha helix. No non-synonymous mutation was detected in the HPV-33 *E7* sequences encoding the alpha helix or the beta sheet.

**Fig 1:**
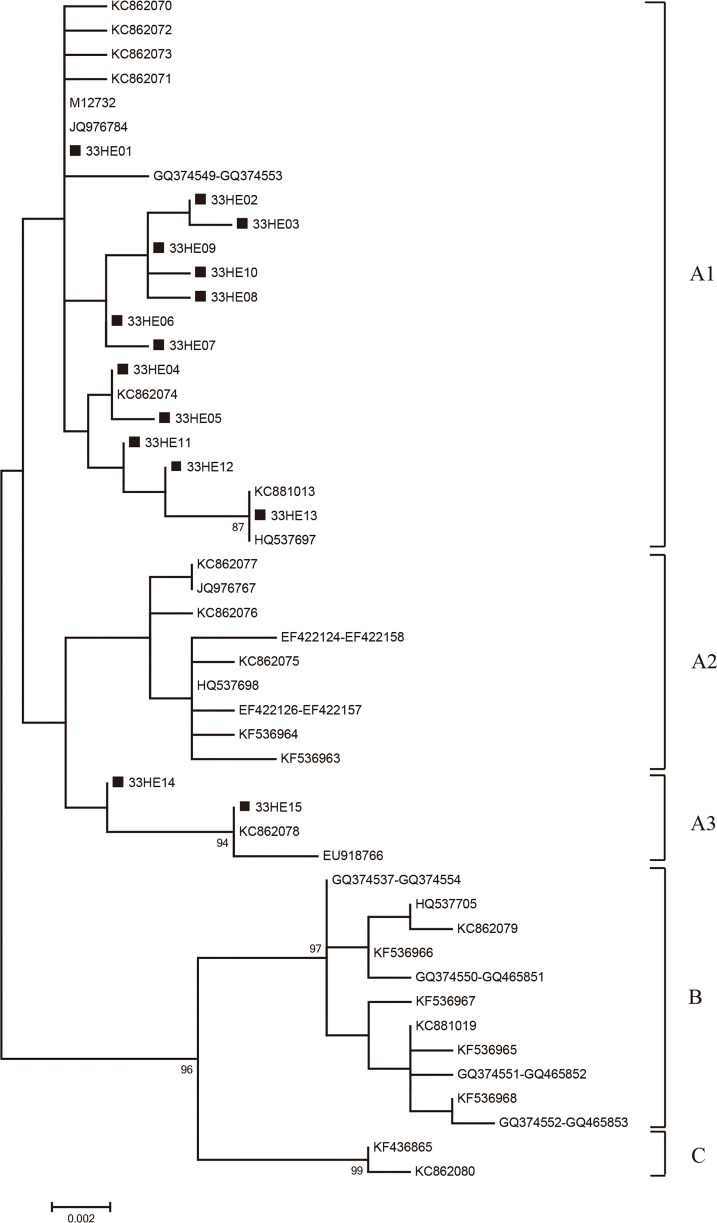
The Maximum-likelihood tree of HPV-33. The Maximum-likelihood tree of HPV-33 variants based on *E6*-*E7* combined sequences. Numbers above the branches indicate the bootstrap values that are greater than 75%.

Among the 216 determinable samples of HPV-33, the A1 and A3 variants were found in 199 (92.13%) and 17 (7.87%) samples, respectively (Table 1 and Fig 1). All of the variants belonged to prototype-like groups (lineage A); whereas no nonprototype-like variant (lineage B) of HPV-33 was observed in our study (Fig 1).

### Gene polymorphism of HPV-58 *E6*/*E7*

When compared with the HPV-58 reference sequences (GenBank: D90400), 11.85% (48/405) of the isolates showed complete *E6*/*E7* sequence homology with the reference, and the nucleotide variation rate of HPV-58 *E6*/*E7* was 88.15% (357/405) among the 405 HPV-58 isolates. Overall, 8 nucleotide sequence variations were detected in the 450-bp *E6* ORF; specifically, 4 mutations were non-synonymous and 4 mutations were synonymous. 13 single nucleotide changes were identified in the 297-bp *E7* ORF, out of which 10 substitutions were non-synonymous and 3 substitutions were synonymous mutations (Table 2). Sequence variability was higher for *E7* than for *E6*. The average probability of a nucleotide sequence deviation from prototype was 20.63 substitutions/10000 bp for *E6* and 78.90 substitutions/10000 bp for *E7*. There were no mutations generating a frame shift or a premature stop codon.

**Table 2:** Nucleotide sequence mutation at *E6/E7* of 17 HPV 58 isolates.

sequence pattern	HPV58 *E6*								HPV58 *E7*													n(405)	sub-lineages
	1	2	2	3	3	3	3	5	5	6	6	7	7	7	7	7	7	7	7	8	8		
	8	0	5	0	6	8	9	4	9	3	9	2	4	5	6	6	6	9	9	0	0		
	7	3	9	7	7	8	5	3	9	2	4	6	4	5	0	1	3	3	8	1	3		
D90400	C	G	A	C	C	A	T	G	G	C	G	T	T	C	G	G	A	A	C	C	T		
58HE01	-	-	-	-	-	-	-	-	-	-	-	-	-	-	-	-	-	-	-	-	-	48	A1
58HE02	T	-	-	-	-	-	-	-	-	-	-	C	-	-	-	-	-	-	-	-	-	7	A1
58HE03	-	-	-	-	-	C	-	-	-	-	-	-	-	-	-	-	-	-	-	-	-	30	A1
58HE04	-	-	-	-	-	C	C	-	-	-	-	-	-	-	-	-	-	-	-	-	-	1	A1
58HE05	-	-	-	-	-	-	-	-	-	-	-	-	G	-	-	-	-	-	-	-	C	1	A1
58HE06	-	-	G	-	-	-	-	-	-	-	-	-	G	-	-	-	-	-	-	-	C	2	A1
58HE07	-	-	-	-	-	C	-	A	-	-	-	-	G	-	-	-	-	-	-	-	C	5	A1
58HE08	-	-	-	-	-	C	-	-	A	-	-	-	G	-	-	-	-	-	-	-	C	13	A1
58HE09	-	-	-	-	-	C	-	-	-	-	-	-	G	-	-	-	-	-	-	-	C	61	A1
58HE10	-	-	-	T	-	-	-	-	-	-		-	G	-	-	A	-	-	-	-	-	1	A2
58HE11	-	-	-	T	-	-	-	A	-	-	A	-	G	-	-	A	-	-	-	-	-	7	A2
58HE12	-	-	-	T	-	-	-	-	-	T	A	-	G	-	-	A	-	-	-	-	-	1	A2
58HE13	-	-	-	T	-	-	-	-	-	-	A	-	G	A	-	A	G	-	-	-	-	26	A2
58HE14	-	-	-	T	-	-	-	-	-	-	A	-	G	-	-	A	-	-	-	-	-	128	A2
58HE15	-	-	-	T	-	-	-	-	-	T	-	-	G	-	A	-	-	-	-	-	-	69	A3
58HE16	-	-	-	T	-	-	-	A	-	T	-	-	G	-	A	-	-	-	-	-	-	4	A3
58HE17	-	C	-	T	A	C	-	-	-	-	-	-	G	-	A	-	-	G	T	A	-	1	B1
aa mutation								R															
		E			D	K		1	R	T	G			T	G	G	T	T		D	V		
	-	3	-	-	8	9	-	4	9	2	4	-	-	6	6	6	6	7	-	7	7		
		2			6	3		5		0	1			1	3	3	4	4		6	7		
		Q			E	N		K	K	I	R			N	S	D	A	A		E	A		
second structure	S	-	S	H	-	-	-	-	-	-	-	-	S	-	-	-	-	H	H	H	H		

The nucleotides conserved with respect to the reference sequence were marked with a dash (-), whereas a variation position was indicated by a letter. The “S” in the last row of the table means Sheet, the “H” means Helix. “n” represents the number of each sequence pattern among the 405 samples examined.

In addition, there were no non-synonymous mutations in the HPV-58 *E6* sequences encoding the alpha helix or the beta sheet; while three non-synonymous mutations (T74A, D76E, and V77A) occurred in HPV-58 *E7* sequences encoding the alpha helix.

**Fig 2:**
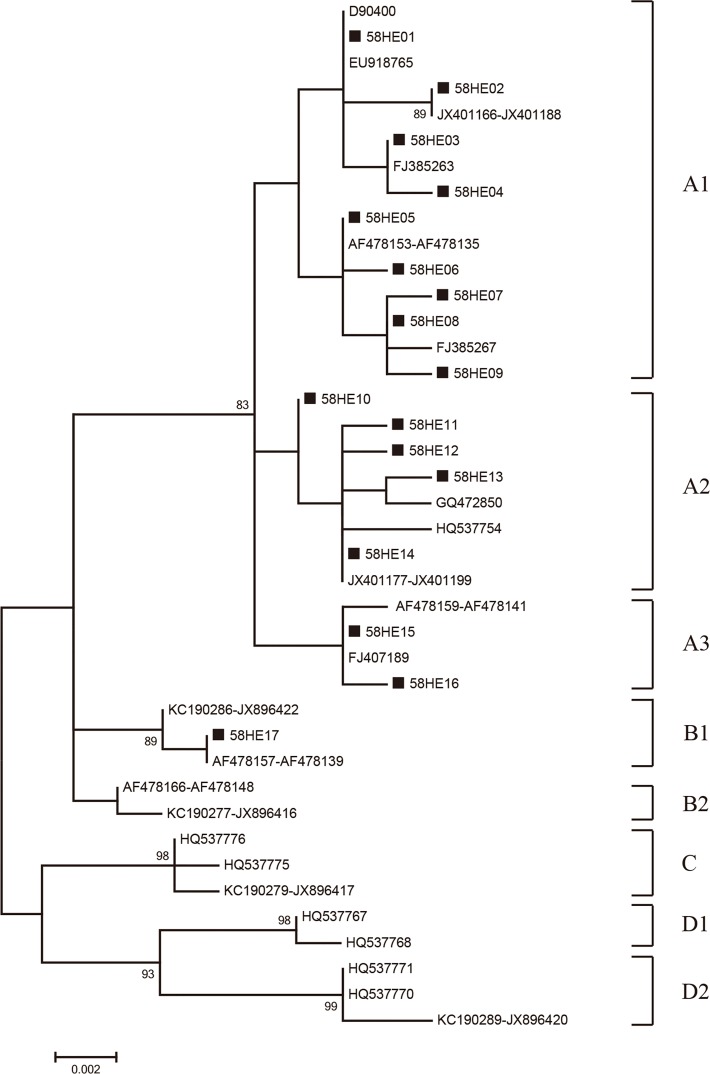
The Maximum-likelihood tree of HPV-58. The Maximum-likelihood tree of HPV-58 variants based on *E6*-*E7* combined sequences. Numbers above the branches indicate the bootstrap values that are greater than 75%.

Among 405 isolates of HPV-58, the A and B variants were found in 404 (99.75%) and 1 (0.25%) isolates, respectively. The nonprototype-like variant (lineage B) of HPV-58 was rare in our study. The sub-lineage A1, A2, and A3 variants were found in 168 (41.48%), 163 (40.25%), and 73 (18.02%) HPV-58 isolates, respectively (Table 2 and Fig 2). Lineages C and D were not observed in the present study (Fig 2).

### Selective pressure analysis and homology comparison

The variable dN/dS ratios were tested among various lineages using the PAML 4.8 software. The results of the selective pressure analysis of HPV-58 and HPV-33 *E6* and *E7* genes (P-value, 0.1) have been summarized in Tables 3 and 4. The positively selected sites for HPV-33 *E6* were K35N, K93N, and R145I; for HPV-33 *E7* were S29T, A45V, A45E, and Q97L; for HPV-58 *E6* were K93N, R145K; and for HPV-58 *E7* were T20I, G41R, G63S, and G63D. The homology comparison results were shown in S1 Fig. K93N and R145 (I/N) of HPV-33 and HPV-58 *E6* were observed at the same two positions.

**Table 3:** Site-specific tests for positive selection on HPV-33 *E6/E7*.

Models	InL	Estimates of parameters	2Δl	*E6* Positively selected sites	*E7* Positively selected sites
M7	-1193.188	p = 0.005, q = 0.047		NA	
M8	-1166.06	p0 = 0.970, p = 0.005, q = 0.012, p1 = 0.030, ω = 86.838	54.256 p<0.01	35K**, 93K**, 145R**	29S**, 45A**,97Q*

**Table 4:** Site-specific tests for positive selection on HPV-58 *E6/E7*.

Models	InL	Estimates of parameters	2Δl	*E6* Positively selected sites	*E7* Positively selected sites
M7	-1273.301	p = 0.005, q = 0.012		NA	
M8	-1256.611	p0 = 0.972, p = 35.347, q = 99.000, p1 = 0.028, ω = 24.138	33.38 p<0.01	93K**, 145R**	20T**, 63G**

Tables 3 and 4: ln L, the log-likelihood difference between the two models; 2Δl, twice the log-likelihood difference between the two models; the positively selected sites were identified with posterior probability ≥ 0.9 using Bayes empirical Bayes (BEB) approach, an asterisk indicates posterior probability ≥ 0.95, and two asterisks indicate posterior probability ≥ 0.99. NA means not allowed. NS means the sites under positive selection, but not reaching the significance level of 0.9.

### Predicted MHC and B-cell epitopes

**Fig 3:**
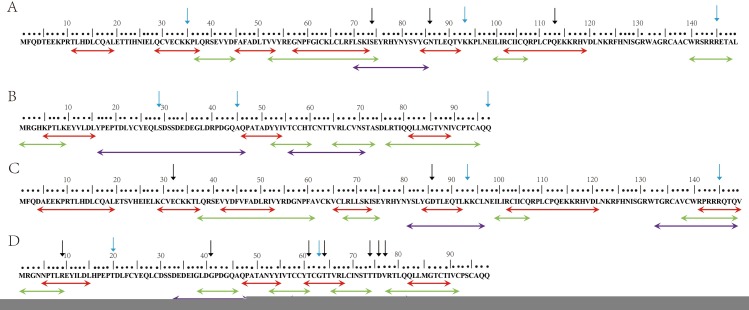
Predicted MHC and B-cell epitopes. Epitopes within the reference sequence of (A) HPV-33 *E6* protein; (B) HPV-33 *E7* protein; (C) HPV-58 *E6* protein; and (D) HPV-58 *E7* protein. Red indicates a consensus of the predicted MHC I epitopes binding not less than 10 HLA I alleles. Green indicates a consensus of the predicted MHC II epitopes binding not less than 10 HLA II alleles. Purple indicates a consensus of the predicted B-cell epitopes of which the score ≥ 0.85 for the combined physical-chemical properties. Black arrow indicates corresponding mutation is only a non-synonymous mutation, and blue arrow indicates corresponding mutation is a non-synonymous mutation and positive selection, simultaneously.

The results of major histocompatibility complex (MHC) epitopes (only epitopes binding not less than 10 HLA class alleles were shown) and B-cell epitopes (only score ≥ 0.85 were shown), as predicted, were summarized in Fig 3, the details were shown in S3–S5 Tables. For MHC I, 8 good epitopes were obtained for HPV-33 *E6*, 5 for HPV-33 *E7*, 11 for HPV-58 *E6* and 5 for HPV-58 *E7*, respectively; For MHC II, 6 good epitopes were obtained for HPV-33 *E6*, 6 for HPV-33 *E7*, 9 for HPV-58 *E6* and 6 for HPV-58 *E7*, respectively; For B-cell, 1 high score epitope was obtained for HPV-33 *E6*, 3 for HPV-33 *E7*, 2 for HPV-58 *E6* and 3 for HPV-58 *E7*, respectively. Amino acids change may influence the epitope’s binding ability, decrease or increase the binding ability, or even lead to disappearance of epitopes or appearance of new epitopes (S3–S5 Tables).

## Discussion

HPV types are divided into different genus according to their biological characteristics. HPV-33 and 58 are known to be closely related to each other, and belong to the α-9 species, which is the principal species consisting of almost all carcinogenic types [44]. The intratypic variations observed in *E6* and *E7* can offer useful information for the distinction and identification of known or new HPV types [45,46]. The present study revealed higher frequencies of HPV-58 *E6*/*E7* variations than HPV-33 *E6*/*E7*. Furthermore, HPV-33 *E7* was observed to be steadier than *E6*; therefore, *E7* was selected as a more suitable target for diagnostic detection of HPV-33 than *E6*. On the contrary, HPV-58 *E6* was steadier than *E7* (χ2 = 16.015, P < 0.01); and was chosen as a more appropriate target for diagnostic detection of HPV-58 than *E7*.

Previous studies revealed two main groups—prototype-like group (lineage A) and nonprototype-like group (lineage B) among HPV-33 variants [27, 29]. However, all of the HPV-33 variants belonged to the prototype-like group, and the nonprototype-like group was not observed in the determinable samples of our study; these findings were similar to the results obtained by Chen et al and Godinez et al [29,30]. The A1 sub-lineage (92.13%) was determined as the major type in southwest China. In a worldwide study on *E6*/*E7* segment of 213 HPV-33 samples, the A2 sub-lineage (59.72%) was the main type in the Asia and Oceania region [29]. However, no A2 sub-lineage was detected in this study. 7.87% of the sub-lineage belonged to A3.

In the current research, the prototype-like group of HPV-58 was the dominating variant; this classification of lineages was in agreement with the results of previous studies [36,47]. Nevertheless, the distribution of HPV-58 variant lineages around China was observed to be different in our study; B1 variant was a nonprototype variant, while the C variant was a nonprototype variant lineage in Hong Kong and Tai Wan [36,47]. When compared with a previous report on HPV-58 in southwest China [33], B lineages were newly found.

The most common mutations observed in HPV-33 *E6* were A231C (K35N, 42/216, a positive mutation) and A387C (K93N, 42/216, a positive mutation); in HPV-33 *E7* were G658C (S29T, 36/216, a positive mutation) and C706A (A45E, 27/216); in HPV-58 *E6* were C307T (237/405) and A388C (K93N 111/405); while the most common non-synonymous mutations in HPV-58 *E7* were T744G (319/405) and G761A (G63D, 163/405, a positive mutation). These mutations may be considered important when *E6*/*E7* are chosen as targets for primer design or diagnostic detection.

To the best of our knowledge, the present study is the first to contrast the HPV-33 and HPV-58 *E6*/*E7* sites under positive selection. The key characteristic of positive selection is that it causes an unusually rapid rise in allele frequency, under positive selection, the positive mutation(s) may rise to high frequency rapidly, help species to adapt to the environments [48]. The selective pressure analysis showed that all the sites that evolved under positive selection were common non-synonymous mutations, indicating that the positively selected variations beneficial for HPV-33 and HPV-58 to accommodate their environments are wide-spread. HPV-33 falls next to HPV-58 in the phylogenetic tree [44]; and remarkably, the positive sites K93N and R145 (I/N) were observed in both HPV-33 and HPV-58 *E6*, they may have evolutionary significance in making HPV-33 and HPV-58 adaptive to their environments. The HPV-58 *E6* mutations T20I and G63S have been reported to increase the risk of developing cervical cancer [36]; interestingly, in the present study, we found that these two mutations were positively selected. Specific intratypic HPV genome variations may be related to virus infectivity, pathogenicity, progression to cervical cancer, viral particle assembly, and host immune response. Of all these variations in the present study, the five newly-reported mutations have been only found in southwest China until now: G329C (S74T) and G542T (R145I, a positive variation) for HPV-33 *E6*, D658C (S29T, a positive variation) for HPV-33 *E7* as well as A259G and T395C for HPV-58 *E6* [24,29,34–36,47,49–51].

Amino acid positions 145–149 form the PDZ binding domain in the *E6* protein; amino acid positions 21–29 form short linear motif responsible for Rb binding in *E7* protein; whereas the positions 58, 61, 91, and 94 act as Zn binding sites in the *E7* protein [29,52]. In the present study, G542T (R145I, a positive mutation) in HPV-33 *E6* and G543A (R145K, a positive mutation) were found at residues 145–149; G658 (S29, a positive mutation) in HPV-33 *E7* was found at residues 21–29, G632T (T20I, a positive mutation) in HPV-58 *E7* was found beside residues 21–29; C755A (T61N) in HPV-58 *E7* was found at residues involved in Zn binding for *E7* protein. Until now, there is no published data to prove that immunity to one variant can prevent another variant in HPV infections [53].

Modern immunoinformatics provide new strategies for the design and identification of antigen-specific epitopic sites that could be used as vaccine targets. The prediction of MHC and B-cell epitope in this study can be potentially used for the vaccine development against specific HPV variants in the Chinese population. In the present study, variations other than HPV-33 *E6* K93N, HPV-33 *E7* Q97L and HPV-58 *E7* T20I were observed in sites belonging to ideal B-cell and/or MHC predicted epitopes. Some variations, like HPV-58 *E6* R145K, occurred at the sites belonging to both B-cell and MHC epitopes; Amino acids change may influence the epitopes, and then the immune recognition of HPV-infected cells. For example, the score of HPV-58 *E6* B-cell epitope 81-96YSLYGDTLEQTLKKCL was 0.90, because of the mutation D86E, the score of epitope 81-96YSLYGETLEQTLNKCL decreased to 0.86; the HPV-58 *E7* predicted epitope 77-85VRTLQQLLM disappeared because of the mutation V77A. However, further experimentation is required to validate the prediction through immunoinformatics.

This is the first study to examine the changes in *E6*/*E7* epitopes of HPV-58 variants in southwest China and to report the variants of HPV-33 *E6*/*E7* in China. The data presented in this study may have significant implications in understanding the intrinsic geographical relatedness and biological differences between HPV-33 and HPV-58 *E6*/*E7*, and may also contribute to the design of clinical diagnostic probes and second-generation therapeutic vaccines based on HPV-33 and HPV-58 *E6*/*E7*.

## Supporting Information

S1 Table HPV-33 reference sequences used in phylogenetic analysis. (DOCX)

S2 Table HPV-58 reference sequences used in phylogenetic analysis. (DOCX)

S3 Table ProPred I analysis for binding of *E6/E7* sequences to HLA class I. (DOCX)

S4 Table ProPred analysis for binding of *E6/E7* sequences to HLA class II. (DOCX)

S5 Table Predicted B-cell epitopes of the *E6/E7* gene. (DOCX)

S1 Fig The homology comparison results of HPV-33 and HPV-58 *E6/E7*. Note: The residues conserved across HPV types were shown in capital letters, whereas the nonconserved residues are given in lowercase letters.
(TIF)
