# Correction: *E6* and *E7* Gene Polymorphisms in Human Papillomavirus Types-58 and 33 Identified in Southwest China

**DOI:** 10.1371/journal.pone.0181475

**Published:** 2017-07-10

**Authors:** Zuyi Chen, Yaling Jing, Qiang Wen, Xianping Ding, Tao Wang, Xuemei Mu, Yuwei Chenzhang, Man Cao

There are errors in the author affiliations. Affiliation 1 and affiliation 3 should not be separated. The affiliations should appear as shown here:

Zuyi Chen^1,2^, Yaling Jing^1,2^, Qiang Wen^1,2^, Xianping Ding^1,2^, Tao Wang^1,2^, Xuemei Mu^1,2^, Yuwei Chenzhang^1,2^, Man Cao^1,2^

**1** Key Laboratory of Bio-Resources and Eco-Environment, Ministry of Education; Institute of Medical Genetics, College of Life Science, Sichuan University, China, **2** Bio-resource Research and Utilization Joint Key Laboratory of Sichuan and Chongqing, Sichuan and Chongqing, China.
