# Supplementary material for: E6 and E7 Gene Polymorphisms in Human Papillomavirus Types-58 and 33 Identified in Southwest China
Source: PLoS One. 2017 Jan 31;12(1):e0171140. doi: 10.1371/journal.pone.0171140 (PMC5283733; doi:10.1371/journal.pone.0171140)
Supplement: S1 Table — (DOCX) [file pone.0171140.s001.docx]

**S1 Table. HPV-33 reference sequences used in phylogenetic analysis.**

| HPV-33 reference sequences (GeneBank accession number) | References |
| --- | --- |
| GQ374549-GQ374553, GQ374537-GQ374554, GQ374550-GQ465851, GQ374551-GQ465852, GQ374552-GQ465853 | Khouadri et al. (2006)[1] |
| EF422126-EF422157, EF422124-EF422158 | Garbuglia et al. (2007)[2] |
| EU918766 | Wu X et al. (2009)[3] |
| HQ537697, HQ537698, HQ537705 | Chen et al.(2011)[4] |
| JQ976784, JQ976767 | J.M. Godínez et al. (2013)[5] |
| KF436865 | Burk et al. (2013)[6] |
| KC862070, KC862072, KC862073, KC862071, KC862074, KC881013, KC862077, KC862076, KC862075, KF536964, KF536963, KC862078, KC862079, KF536966, KF536967, KC881019, KF536965, KF536968 , KC862080 | Chen AA et al. (2014)[7] |

References

1. Khouadri S, Villa LL, Gagnon S, Koushik A, Richardson H, Ferreira S, et al. Human papillomavirus type 33 polymorphisms and high-grade squamous intraepithelial lesions of the uterine cervix. J Infect Dis. 2006;194: 886-894.

2. Garbuglia AR, Carletti F, Minosse C, Piselli P, Zaniratti MS, Serraino D, et al. Genetic variability in E6 and E7 genes of human papillomavirus -16, -18, -31 and -33 from HIV-1-positive women in Italy. New Microbiol. 2007;30: 377-382.

3. Wu X, Zhang C, Feng S, Liu C, Li Y, Yang Y, et al. Detection of HPV types and neutralizing antibodies in Gansu province, China. J Med Virol. 2009;81: 693-702.

4. Chen Z, Schiffman M, Herrero R, Desalle R, Anastos K, et al. Evolution and taxonomic classification of human papillomavirus 16 (HPV16)-related variant genomes: HPV31, HPV33, HPV35, HPV52, HPV58 and HPV67. PLoS One. 2011;6: e20183.

5. Godínez JM, Heideman DA, Gheit T, Alemany L, Snijders PJ, Tommasino M, et al. Differential presence of Papillomavirus variants in cervical cancer: an analysis for HPV33, HPV45 and HPV58. Infect Genet Evol. 2013;13: 96-104.

6. Burk RD, Harari A, Chen Z. Human papillomavirus genome variants. Virology. 2013;445: 232-243.

7. Chen AA, Heideman DA, Boon D, Chen Z, Burk RD, De Vuyst H, et al. Human papillomavirus 33 worldwide genetic variation and associated risk of cervical cancer. Virology. 2014;448: 356-362.
