# Supplementary material for: E6 and E7 Gene Polymorphisms in Human Papillomavirus Types-58 and 33 Identified in Southwest China
Source: PLoS One. 2017 Jan 31;12(1):e0171140. doi: 10.1371/journal.pone.0171140 (PMC5283733; doi:10.1371/journal.pone.0171140)
Supplement: S2 Table — (DOCX) [file pone.0171140.s002.docx]

**S2 Table. HPV-58 reference sequences used in phylogenetic analysis.**

| HPV-58 reference sequences (GeneBank accession number) | References |
| --- | --- |
| AF478153-AF478135, AF478159-AF478141, AF478157-AF478139, AF478166-AF478148 | Chan PK et.al.(2002)[1] |
| FJ385263, FJ385267, FJ407189 | Wu EQ et.al.(2009)[2] |
| HQ537767, HQ537768, HQ537771, HQ537770, HQ537776, HQ537775, HQ537754, GQ472850, D90400, EU918765 | Chen. Z et.al.(2011)[3] |
| KC190286-JX896422, KC190277-JX896416, KC190289-JX896420, KC190279-JX896417 | Chan PK et.al.(2013)[4] |
| JX401166-JX40118, JX401177-JX401199 | Yang Let.al.(2014)[5] |

References

1. Chan PK, Lam CW, Cheung TH, Li WW, Lo KW, Chan MY, et al. Association of human papillomavirus type 58 variant with the risk of cervical cancer. J Natl Cancer Inst. 2002;94: 1249-1253.

2. Wu EQ, Zha X, Yu XH, Zhang GN, Wu YG, Fan Y, et al. Profile of physical status and gene variation of human papillomavirus 58 genome in cervical cancer. J Gen Virol. 2009;90: 1229-1237.

3. Chen Z, Schiffman M, Herrero R, Desalle R, Anastos K, Segondy M, et al. Evolution and Taxonomic Classification of Human Papillomavirus 16 (HPV16)-Related Variant Genomes:HPV31, HPV33, HPV35, HPV52, HPV58 and HPV67. PLoS One. 2011;6: e20183.

4. Chan PK, Zhang C, Park JS, Smith-McCune KK, Palefsky JM, Giovannelli L, et al. Geographical distribution and oncogenic risk association of human papillomavirus type 58 E6 and E7 sequence variations. Int J Cancer. 2013;132: 2528-2536.

5. Yang L, Yang H, Chen J, Huang X, Pan Y, Li D, et al. Genetic variability of HPV-58 E6 and E7 genes in Southwest China. Infect Genet Evol. 2014;21: 395-400.
