# Supplementary material for: E6 and E7 Gene Polymorphisms in Human Papillomavirus Types-58 and 33 Identified in Southwest China
Source: PLoS One. 2017 Jan 31;12(1):e0171140. doi: 10.1371/journal.pone.0171140 (PMC5283733; doi:10.1371/journal.pone.0171140)
Supplement: S3 Table — (DOCX) [file pone.0171140.s003.docx]

**S3 Table. ProPred I analysis for binding of *E6/E7* sequences to HLA class I.**

| Protein | Reference sequence | | | | Variants sequence | | | |
| --- | --- | --- | --- | --- | --- | --- | --- | --- |
|  | Start | End | Epitope sequence | No.of binding alleles (MHC I) | Start | End | Epitope sequence | No.of binding alleles (MHC I) |
| HPV33 *E6* | 11 | 19 | TLHDLCQAL | 18/47 | 11 | 19 | TLHDLCQAL | 18/47 |
|  | 29 | 37 | QCVECK**K**PL | 13/47 | 29 | 37 | QCVECK**N**PL | 13/51 |
|  | 45 | 53 | FAFADLTVV | 13/47 | 45 | 53 | FAFADLTVV | 13/47 |
|  | 57 | 65 | GNPFGICKL | 10/47 | 57 | 65 | GNPFGICKL | 10/47 |
|  | 65 | 73 | LCLRFLSKI | 12/47 | 65 | 73 | LCLRFLSKI | 12/47 |
|  | 84 | 92 | YG**N**TLEQTV | 10/47 | 84 | 92 | YG**H**TLEQTV | 10/47 |
|  |  |  |  |  | 88 | 96 | LEQTV**N**KPL | 11/47 |
|  | 102 | 110 | RCIICQRPL | 10/47 | 102 | 110 | RCIICQRPL | 10/47 |
|  | 111 | 119 | CP**Q**EKKRHV | 13/47 | 111 | 119 | CP**R**EKKRHV | 13/47 |
|  |  |  |  |  | 141 | 149 | RSRR**I**ETAL | 10/47 |
| HPV33 *E7* | 5 | 13 | KPTLKEYVL | 20/47 | 5 | 13 | KPTLKEYVL | 20/47 |
|  | 7 | 15 | TLKEYVLDL | 11/47 | 7 | 15 | TLKEYVLDL | 11/47 |
|  | 14 | 22 | DLYPEPTDL | 11/47 | 14 | 22 | DLYPEPTDL | 11/47 |
|  | 46 | 54 | QPATADYYI | 11/47 | 46 | 54 | QPATADYYI | 11/47 |
|  | 81 | 90 | QLLMGTVNI | 11/47 | 81 | 90 | QLLMGTVNI | 11/47 |
| HPV58 *E6* | 4 | 12 | DAEEKPRTL | 12/47 | 4 | 12 | DAEEKPRTL | 12/47 |
|  | 11 | 19 | TLHDLCQAL | 18/47 | 11 | 19 | TLHDLCQAL | 18/47 |
|  | 29 | 37 | KCV**E**CKKTL | 14/47 | 29 | 37 | KCV**Q**CKKTL | 12/47 |
|  | 42 | 50 | VYDFVFADL | 10/47 | 42 | 50 | VYDFVFADL | 10/47 |
|  | 45 | 53 | FVFADLRIV | 11/47 | 45 | 53 | FVFADLRIV | 11/47 |
|  | 65 | 73 | VCLRLLSKI | 10/47 | 65 | 73 | VCLRLLSKI | 10/47 |
|  | 84 | 92 | YG**D**TLEQTL | 13/47 | 84 | 92 | YG**E**TLEQTL | 13/47 |
|  |  |  |  |  | 88 | 96 | LEQTL**N**KCL | 11/47 |
|  | 102 | 110 | RCIICQRPL | 10/47 | 102 | 110 | RCIICQRPL | 10/47 |
|  | 111 | 119 | CPQEKKRHV | 13/47 | 111 | 119 | CPQEKKRHV | 13/47 |
|  | 113 | 121 | QEKKRHVDL | 10/47 | 113 | 121 | QEKKRHVDL | 10/47 |
|  | 141 | 149 | RPRR**R**QTQV | 13/47 | 141 | 149 | RPRR**K**QTQV | 13/47 |
| HPV58 *E7* | 5 | 13 | NPTL**R**EYIL | 18/47 | 5 | 13 | NPTL**K**EYIL | 17/47 |
|  | 7 | 15 | TL**R**EYILDL | 11/47 | 7 | 15 | TL**K**EYILDL | 11/47 |
|  | 47 | 55 | QPATANYYI | 12/47 | 47 | 55 | QPATANYYI | 12/47 |
|  | 60 | 68 | Y**T**C**GT**TVRL | 12/47 | 60 | 68 | YTC**S**TTVRL | 10/47 |
|  |  |  |  |  | 60 | 68 | YTC**D**TTVRL | 14/47 |
|  |  |  |  |  | 60 | 68 | Y**N**C**DA**TVRL | 11/47 |
|  | 82 | 90 | QLLMGTCTI | 11/47 | 82 | 90 | QLLMGTCTI | 11/47 |

Note: Sequences with amino acids change were highlight in red.
