# Supplementary material for: E6 and E7 Gene Polymorphisms in Human Papillomavirus Types-58 and 33 Identified in Southwest China
Source: PLoS One. 2017 Jan 31;12(1):e0171140. doi: 10.1371/journal.pone.0171140 (PMC5283733; doi:10.1371/journal.pone.0171140)
Supplement: S4 Table — (DOCX) [file pone.0171140.s004.docx]

**S4 Table. ProPred analysis for binding of *E6/E7* sequences to HLA class II.**

| Protein | Reference sequence | | | | Variants sequence | | | |
| --- | --- | --- | --- | --- | --- | --- | --- | --- |
|  | Start | End | Epitope sequence | No. of binding alleles (MHC II) | Start | End | Epitope sequence | No. of binding alleles (MHC II) |
| HPV33 *E6* | 37 | 45 | LQRSEVYDF | 12/51 | 37 | 45 | LQRSEVYDF | 12/51 |
|  | 52 | 60 | VVYREGNPF | 13/51 | 52 | 60 | VVYREGNPF | 13/51 |
|  | 60 | 68 | FGICKLCLR | 10/51 | 60 | 68 | FGICKLCLR | 10/51 |
|  | 67 | 75 | LRFLSKI**S**E | 34/51 | 67 | 75 | LRFLSKI**T**E | 34/51 |
|  | 99 | 107 | ILIRCIICQ | 28/51 | 99 | 107 | ILIRCIICQ | 28/51 |
|  | 140 | 148 | WRSRR**R**ETA | 11/51 | 140 | 148 | WRSRR**I**ETA | 11/51 |
| HPV33 *E7* | 1 | 9 | MRGHKPTLK | 20/51 | 1 | 9 | MRGHKPTLK | 20/51 |
|  | 52 | 60 | YYIVTCCHT | 13/51 | 52 | 60 | YYIVTCCHT | 13/51 |
|  | 65 | 73 | VRLCVNSTA | 19/51 | 65 | 73 | VRLCVNSTA | 19/51 |
|  | 76 | 84 | LRTIQQLLM | 28/51 | 76 | 84 | LRTIQQLLM | 28/51 |
|  | 82 | 90 | LLMGTVNIV | 11/51 | 82 | 90 | LLMGTVNIV | 11/51 |
|  | 87 | 95 | VNIVCPTCA | 16/51 | 87 | 95 | VNIVCPTCA | 16/51 |
| HPV58 *E6* |  |  |  |  | 26 | 34 | IELKCV**Q**CK | 10/51 |
|  |  |  |  |  | 31 | 39 | V**Q**CKKTLQR | 20/51 |
|  | 37 | 45 | LQRSEVYDF | 12/51 | 37 | 45 | LQRSEVYDF | 12/51 |
|  | 45 | 53 | FVFADLRIV | 26/51 | 45 | 53 | FVFADLRIV | 26/51 |
|  | 50 | 58 | LRIVYRDGN | 11/51 | 50 | 58 | LRIVYRDGN | 11/51 |
|  | 52 | 60 | IVYRDGNPF | 13/51 | 52 | 60 | IVYRDGNPF | 13/51 |
|  | 53 | 61 | VYRDGNPFA | 11/51 | 53 | 61 | VYRDGNPFA | 11/51 |
|  | 67 | 75 | LRLLSKISE | 36/51 | 67 | 75 | LRLLSKISE | 36/51 |
|  | 99 | 107 | ILIRCIICQ | 28/51 | 99 | 107 | ILIRCIICQ | 28/51 |
|  | 138 | 145 | VCWRPRR**R**Q | 12/51 | 138 | 145 | VCWRPRR**K**Q | 12/51 |
|  | 140 | 148 | WRPRR**R**QTQ | 18/51 | 140 | 148 | WRPRR**K**QTQ | 24/51 |
| HPV58 *E7* | 1 | 9 | MRGNNPTL**R** | 10/51 | 1 | 9 | MRGNNPTL**K** | 17/51 |
|  | 37 | 45 | IGLD**G**PDGQ | 10/51 | 37 | 45 | IGLD**R**PDGQ | 10/51 |
|  | 53 | 61 | YYIVTCCY**T** | 13/51 | 53 | 61 | YYIVTCCY**N** | 13/51 |
|  | 66 | 74 | VRLCINST**T** | 13/51 | 66 | 74 | VRLCINST**A** | 19/51 |
|  | 77 | 85 | **V**RTLQQLLM | 34/81 |  |  |  |  |
|  | 84 | 92 | LMGTCTIVC | 12/51 | 84 | 92 | LMGTCTIVC | 12/51 |

Note: Sequences with amino acids change were highlight in red.
