# Supplementary material for: E6 and E7 Gene Polymorphisms in Human Papillomavirus Types-58 and 33 Identified in Southwest China
Source: PLoS One. 2017 Jan 31;12(1):e0171140. doi: 10.1371/journal.pone.0171140 (PMC5283733; doi:10.1371/journal.pone.0171140)
Supplement: S5 Table — (DOCX) [file pone.0171140.s005.docx]

**S5 Table. Predicted B-cell epitopes of the *E6/E7* gene.**

| Protein | Reference sequence | | | | Variants sequence | | | |
| --- | --- | --- | --- | --- | --- | --- | --- | --- |
|  | Rank | Sequence | Start position | Score | Rank | Sequence | Start position | Score |
| HPV33 *E6* | 1 | LSKI**S**EYRHYNYSVYG | 70 | 0.85 | 1 | LSKI**T**EYRHYNYSVYG | 70 | 0.85 |
| HPV33 *E7* | 1 | SSDEDEGLDRPDGQ**A**Q | 31 | 0.86 |  |  |  |  |
|  | 2 | TCCHTCNTTVRLCVNS | 56 | 0.85 | 1 | TCCHTCNTTVRLCVNS | 56 | 0.85 |
|  | 2 | YPEPTDLYCYEQL**S**DS | 16 | 0.85 |  |  |  |  |
| HPV58 *E6* | 1 | YSLYG**D**TLEQTLKKCL | 81 | 0.9 | 2 | YSLYG**E**TLEQTLNKCL | 81 | 0.86 |
|  | 2 | TGRCAVCWRPRR**R**QTQ | 133 | 0.89 | 1 | TGRCAVCWRPRR**K**QTQ | 133 | 0.88 |
| HPV58 *E7* | 1 | TCCY**T**C**GT**TVRLCINS | 57 | 0.89 |  |  |  |  |
|  | 2 | RLCINST**T**T**DV**RTLQQ | 67 | 0.88 |  |  |  |  |
|  | 3 | DEDEIGLD**G**PDGQAQP | 33 | 0.86 | 1 | DEDEIGLD**R**PDGQAQP | 33 | 0.91 |
|  |  |  |  |  | 2 | ATANYYIVTCCYNC**DA** | 49 | 0.87 |

Note: Sequences with amino acids change were highlight in red. High score means good binder.
