# Supplementary figures and images for: E6 and E7 Gene Polymorphisms in Human Papillomavirus Types-58 and 33 Identified in Southwest China
Source: PLoS One. 2017 Jan 31;12(1):e0171140. doi: 10.1371/journal.pone.0171140 (PMC5283733; doi:10.1371/journal.pone.0171140)

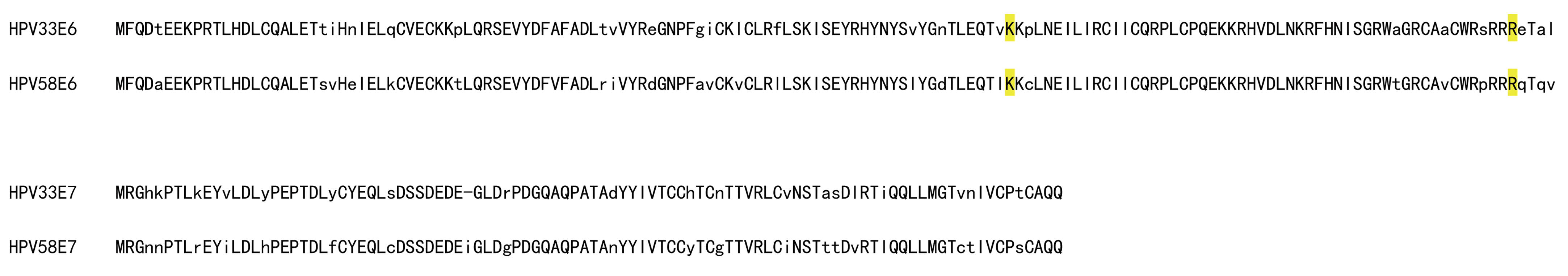

Supplement: S1 Fig — Note: The residues conserved across HPV types were shown in capital letters, whereas the nonconserved residues are given in lowercase letters. (TIF) [file pone.0171140.s006.tif]
